# A Systematic Review of the Causes and Management of Nonthrombotic Embolic Stroke of Tissue Origin

**DOI:** 10.1155/2018/8092862

**Published:** 2018-04-24

**Authors:** Sarah Mello, Ciaran Judge, Roisin Kelly, David Bradley, Joseph Harbison

**Affiliations:** ^1^Acute Stroke Service, St James Hospital, Dublin, Ireland; ^2^Trinity College Institute for Neurosciences, Trinity College, University of Dublin, Dublin, Ireland

## Abstract

**Introduction:**

Various bodily tissues have been reported to enter the arterial circulation and embolize to the brain resulting in ischemic stroke. Most frequently nonthrombotic embolic stroke (NTES) of tissue origin is iatrogenic or related to an underlying disease process. With the increase in elective surgery and intravascular procedures, NTES may increase in prevalence.

**Aim:**

To compile a summary of the background, incidence, presentation, and treatment of NTES of tissue origin, by conducting a systematic review of the current literature.

**Summary of Review:**

We searched EMBASE and MEDLINE for articles on NTES of tissue origin published in English with no restriction on publication date (search date June 2017). 800 articles were identified and screened and 159 articles were ultimately reviewed in full text and included in qualitative analysis. Articles deemed relevant were assessed by a second reviewer to confirm compatibility with the inclusion criteria. References of included articles were reviewed for relevant publications. We categorized the pathology of the emboli into the following groups: amniotic fluid (4 publications), tumour (60 publications), fat (43 publications), cholesterol (19 publications), and intravascular debris (12 publications). We then summarized the available literature on each cause of NTES.

**Conclusions:**

NTES of tissue origin is an uncommon but important diagnosis to consider particularly in younger stroke patients and in certain clinical settings. Treatment for NTES is currently anecdotal and based on small case series. Embolectomy may emerge as the therapy of choice due to the longer treatment timeframe and heterogeneity of the emboli.

## 1. Introduction

Although embolic strokes are most commonly thrombotic in nature, they can also arise from other sources. Any bodily tissue entering into the arterial circulation poses the potential to cause cerebral ischemia. Patients with direct links from the venous to arterial circulation, such as those with cardiac septal defects, are more at risk of this phenomenon. Patients with nonthrombotic embolic stroke (NTES) can present similarly to those with commoner emboli, yet diagnosis and treatment may be delayed due to a lack of awareness of the pathological processes.

We reviewed the available literature on NTES of tissue source to create a review of the pathophysiology, causes, and treatment options available for these cases.

## 2. Methods

An initial literature review was performed to identify potential causes of NTES then a formal systematic review was conducted to identify all references. References were identified by searching EMBASE and MEDLINE databases between January 1956 and June 2017. Only articles written in or translated into English were included. Case series and case reports were included if they discussed cases involving primary diagnoses of embolic cerebrovascular stroke or transient ischemic attacks arising from matter other than thrombus.

**Figure 1:**
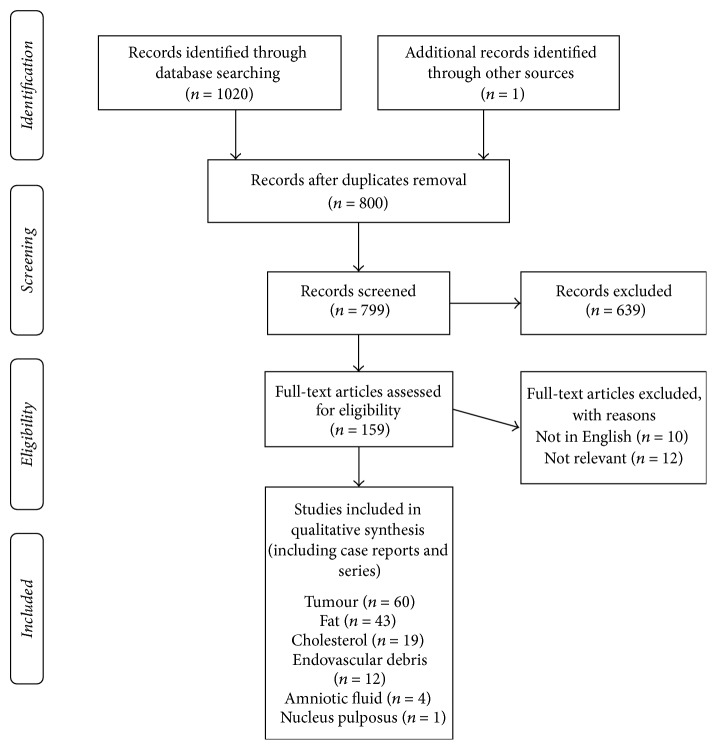
PRIMSA 2009 flow diagram.

A PRISM diagram of the search strategy is included in Figure 1. Search terms were added over time as it became apparent that words such as “debris” would yield more results. All search results were screened by analysis of the title and abstracts, and any potentially relevant articles were read in full text to assess suitability. Articles deemed relevant were then independently assessed by a second reviewer in a nonblinded manner to confirm compatibility with the inclusion criteria. Any disagreements were resolved by consensus. Excluded articles were entered along with the included results into the PRISMA Flow Sheet to summarize the selection process. There was limited risk of bias as we were searching case reports and case series. We divided the included studies into the following groups based on the pathology of the embolus: amniotic fluid, tumour, fat, cholesterol, and intravascular debris. All data accessed for the study has been submitted to the journal either in the article proper or in Appendix 1.

Emboli resulting from infectious sources, for example, from vegetation in infectious endocarditis, were not included as many of these contain a thrombotic component.

## 3. Amniotic Fluid Embolism

### 3.1. Background and Incidence

Amniotic fluid embolism (AFE) is an uncommon (8 : 100,000 deliveries) but often lethal complication of childbirth. Amniotic fluid, fetal cells, and other debris can enter the maternal venous circulation through the endocervical veins or the placental bed causing cardiorespiratory failure and disseminated intravascular coagulation (DIC) resulting in death or permanent neurological disability in up to two-thirds of patients [1]. The exact cause of AFE is not known, but associated risk factors include advanced maternal age, placenta previa, and caesarean delivery [2].

### 3.2. Presentation and Treatment

Cerebral infarction resulting from AFE is such a rare occurrence that our literature review revealed four publications including two case reports [3, 4]. In both cases, the mother was under 35 and the embolism was paradoxical, crossing into the arterial circulation either through a patent foramen ovale or through an atrial septal defect. The AFE occurred during the early stages of labour after rupture of the membranes, but prior to active labour. Raised intrathoracic pressure secondary to abdominal muscle contractions may have caused a left to right cardiac shunt to temporarily reverse, allowing the AFE to cross from the venous to the arterial circulation.

On presentation, these patients were extremely unwell suffering from collapse, seizures, DIC, and cardiorespiratory arrest. The goals of treatment initially centered on stabilizing the mother and safely delivering the foetus. Stroke symptoms were noticed one and five days following initial presentation when the patient was stable enough to be extubated. In one case a single large infarction occurred in the left middle cerebral artery territory and in the other case smaller bilateral infarcts occurred throughout multiple vascular territories. Both mothers and babies recovered well and were discharged home with minimal neurological deficits.

## 4. Tumour Embolism

### 4.1. Background

Cerebral infarction secondary to malignancy is a well-documented phenomenon. Stroke can occur from brain metastasis and surrounding vasogenic oedema, tumour emboli, or hemorrhage from aneurysms. In order to cause cerebral infarction, the tumour must gain access to the arterial circulation. Thus of 60 publications identified most reported cases of tumour embolism involved primary cardiac malignancies or lung malignancies with invasion of the pulmonary vein. More rarely, cases have been reported of cardiac metastases, breast cancer, and primary arterial tumour resulting in stroke. Tumour emboli have also been reported to travel through cardiac defects such as a patent foramen ovale or ventricular septal defect or to embolize during surgery.

### 4.2. Incidence

Cardiac myxoma is the most common cause of tumour embolism to the brain in addition to being a thrombotic source and trigger for atrial fibrillation. Myxomas affect the left atrium in 80% of cases, and myxomas with mobile elements and a villous surface are more likely to embolize regardless of the size of the tumour. Case series have demonstrated cerebral embolism in 20–30% of patients with cardiac myxomas [5]. Most cases of stroke relating to myxoma are ischemic (80%), but hemorrhagic strokes have been described as well. Cardiac sarcoma is the most common malignant tumour of the heart but is rare with a reported incidence of 0.001–0.03% that only four case reports documenting tumour embolism in these patients were identified [6].

In a large postmortem study cardiac metastases were reported in 9% of cases, often these remained undetected and asymptomatic during life. The most common metastases were from malignant melanoma, lung, and breast tumours [7]. The latter two invade the pulmonary veins and left atrium through direct extension, whereas melanoma spreads to the heart via the haematogenous route. Resultant tumour embolism is rare, with our literature review revealing three cases of tumour embolism from myocardial metastases and thirteen cases from direct cardiac invasion.

New neurological deficits may be the primary presenting complaint in patients with underlying malignancy. Of the 43 case reports of tumour embolism identified in this review, 22 (51%) report stroke as being the first presentation of malignancy. In one case series of 74 patients, atrial myxoma first presented as stroke in 16% of patients [8]. In other instances, the patient may initially present with constitutional symptoms relating to the malignancy itself. Stroke follows then from tumour reoccurrence, extension to the arterial vasculature, or embolization during surgery.

Patients with tumour embolism tend to be younger than the typical stroke population. The mean age of patients in this review was 52.5 (range 3–83 +/− 22.6). It is important to consider occult malignancy in younger patients presenting with cryptogenic stroke. Studies suggest that myxomas occur in up to 0.5% of all stroke patients with women in their fifties at the greatest risk [9].

Cardiac tumours are best evaluated by transthoracic echocardiography. The definitive treatment for patients with atrial myxoma is cardiac surgery to remove the tumour. These patients do well with a survival rate similar to that of the general population. Tumour reoccurrence is low and occurs in around 13% of patients within four years of surgery [10].

### 4.3. Treatment

Treatment of acute stroke secondary to tumour embolus is anecdotal and based on a few reported case studies. Both thrombolysis and embolectomy have been trialed with varying success rates. Most of the case reports are of patients with myxoma emboli, but there are also reports on acute stroke treatment in cardiac sarcoma, lung, and breast tumour emboli. Emboli, particularly those associated with myxomas, can consist of pure tumour or may have a thrombotic component. When considering thrombolysis in patients with tumour emboli it is important to bear in mind the increased risk of hemorrhage [8]. Tumour emboli are more prone to be associated with haemorrhagic transformation than thrombi due to the friable nature of malignant cells [10]. Cerebral metastases or malignancy associated aneurysms may also be present which increase the risk of hemorrhage. This review found ten cases of tumour emboli from cardiac myxoma that have been treated with thrombolysis. Two cases were given intra-arterial urokinase, one case had intra-arterial rt-PA, and seven cases had intravenous rt-PA. Among these ten cases, six had clinical improvement, two did not respond to treatment, one went on to have an embolectomy, and one deteriorated due to bleeding complications.

Nine case reports and series were found in which tumour embolism was treated with embolectomy. Four of these cases were in patients with myxoma, one cardiac sarcoma, one breast tumour, one lung tumour, and one case of melanoma. Various types of thrombectomy devices were employed but no comment can be made as to which was most effective.

## 5. Fat Embolism

### 5.1. Background

Fat embolism syndrome (FES) refers to the classical triad of respiratory distress, neurological impairment, and petechial rash that occur when fat microglobules enter the systemic circulation. The basis for diagnosis of FES is described in the Gurd and Wilson criteria (Table 1). However, among 60 publications identified there were several cerebral fat embolism (CFE) cases described in which the pulmonary and cutaneous manifestations of FES are absent. CFE can prove a diagnostic challenge as no gold standard exists for the diagnosis and the neurological manifestations vary widely. Patients with CFE can present with coma, seizure, focal neurological deficits, or cognitive decline.

The pathophysiology of FES involves both mechanical and biochemical pathways. Fat microglobules released from bone marrow or other fat stores during trauma or surgery gain access to the circulation through venous sinusoids. The fat globules initiate an inflammatory response and create a prothrombotic state causing platelet aggregation [11]. The microglobules can gain access to the arterial circulation via cardiac defects or directly through the pulmonary capillary bed if they are less than 5 um in size [12]. Once in the cerebral vasculature, the fat globules cause ischemia as well as vasogenic and cytotoxic edema. Table 1 outlines the Gurd and Wilson criteria for diagnosis of fat embolism [13]. Diagnosis of FES requires the presence of at least one major criterion and at least four minor criteria.

### 5.2. Incidence

Long bone fractures and orthopaedic surgery are the most common causes of FES and CFE. The release of fat globules into the blood stream during orthopaedic procedures is high, with most patients exhibiting transient hypoxemia or neurological dysfunction perioperatively. One study detected fat globules in the serum of 67% of orthopaedic trauma patients [13]. However, the clinical incidence of FES in patients with long bone or pelvic fractures remains at around 0.9–11% [14]. In cases of multiple trauma, this figure is much higher.

CFE has been associated with various other traumatic and nontraumatic causes (Table 2). These causes are so rare that the true incidence in not known. Patients with cardiac shunts, diabetes, or intrinsic muscle, bone, or liver disease are at higher risk of CFE.

### 5.3. Presentation

Our literature review revealed 49 case reports of CFE. Presentation varied widely from the classical triad of FES, to isolated cerebral deficits. Neurological changes in cases of CFE vary widely, as mentioned previously, and can prove diagnostically challenging, especially when not accompanied by respiratory and dermatological involvement. In a few but not all cases, patients had cardiac septal deficits or a pulmonary arteriovenous malformation detected to permit paradoxical embolization.

In orthopaedic causes of FES, symptoms generally appear gradually 12–36 hours after fat globule are dispersed into the circulation [15]. However, FES can also occur intraoperatively with patients failing to wake up from anaesthesia, or respiratory failure with failure to extubate. Respiratory manifestations of FES occur in up to 40% of patients and skin changes in up to 60% [15]. Neurological involvement is also very common.

Autologous fat injection a technique used in augmentation of soft tissues such as in Breast Reconstruction may result in the inadvertent introduction of lipid directly into the arterial circulation. Thus, focal neurological symptoms, especially retinal symptoms, occur rapidly following injection, and other systemic manifestations of CFE are frequently absent.

Most reported patients with FES made a good recovery and are discharged with a favourable functional outcome. The circulating fat globules tend to be small, less than a millimeter in size. They are only able to occlude smaller arteries and exert most of their damage by initiating an inflammatory response. This usually settles down with conservative management. If a large cerebral artery is not occluded and respiratory status is not compromised, the outlook tends to be favourable.

### 5.4. Imaging

CFE produces characteristic changes on both computed tomography (CT) and magnetic resonance imaging (MRI). Often times, CT brain imaging is normal in patients with CFE; however a “hypodense artery sign” may be present where the fatty embolus can be seen in a large proximal artery with a Houndsfield unit measurement of −100 to −50, indicating an intraluminal fat embolism [16] in contrast to the hyperdense artery commonly found in thromboembolic stroke.

MRI has proved diagnostically useful in detecting the intracerebral changes of CFE. A systematic review by Kuo et al. described the changing imaging patterns along the disease time course [17]. The most common pattern seen in the acute stage of CFE is scattered cytotoxic edema, known as the starfield pattern. This is relatively nonspecific, and MRI shows scattered spot lesions with restricted diffusion on diffusion weighted imaging (DWI) sequences. In CFE, these lesions tend to be distributed bilaterally in the deep grey matter and watershed zones. Petechial hemorrhages were detected in 60% of cases.

### 5.5. Treatment

Currently no disease specific treatment exists for FES, and the cornerstone of treatment centers around supportive care. Multiple studies have been done examining the use of corticosteroids and heparin in the treatment and prevention of FES, but they have not been able to definitively prove a benefit [11].

Our search detected one case report on embolectomy used to treat CFE. This report details the case of new neurological symptoms occurring in the acute postoperative period following hip revision surgery. The CT brain scan revealed a hypodense right middle cerebral artery and embolectomy was performed using a stent retriever device. The artery was successfully recanalized; however, the patient had hemorrhagic transformation of the infarction shortly thereafter resulting in a poor outcome [18].

## 6. Cholesterol Embolism

### 6.1. Background

Cholesterol embolism syndrome (CES) refers to embolization of cholesterol crystals from a proximal large artery to distal smaller caliber arterial beds resulting in inflammation and ischemia. CES is usually characterized by showers of microemboli occurring over a period of time [19]. CES can affect multiple organ systems commonly causing acute renal failure, livedo reticularis, and eosinophilia through activation of an acute inflammatory response. Cerebral ischemia can ensue if embolization occurs from a plaque in the ascending aorta, aortic arch, or internal carotid arteries. Experiments using rat models have demonstrated more severe brain tissue necrosis following cholesterol emboli as compared to thromboemboli [20]. We identified 19 publications including case reports describing this phenomenon.

### 6.2. Incidence

The incidence of stroke caused by cholesterol embolism is not known but occurs more often in patients with a heavy intra-arterial plaque burden, typically older male smokers. Risk factors for CES include cardiac catheterization, cardiac surgery, carotid interventions, thrombolysis, and anticoagulation, although it can also occur spontaneously. In an autopsy study of patients who had undergone myocardial revascularization surgery, it was found that 16% had cerebral cholesterol emboli [21]. Analysis of embolic debris captured in distal protection filters shows cholesterol emboli in 36% of cases after carotid stenting [22] and 30% of patients with transaortic valve replacements [23]. To date, CES has not been included as a recognized complication in any of the large thrombolysis trials, so its incidence relating to this therapy is not known.

### 6.3. Presentation

Our literature review revealed 12 case reports of stroke, transient ischemic attack, or retinal artery occlusion as a result of cholesterol emboli. The patients' age ranged from 49 to 80 years and 9 of the 12 were male. In four cases, patients initially presented with neurological deficits due to thromboembolic stroke. These patients went on to receive either thrombolysis or warfarin therapy that caused new neurological symptoms and clinical deterioration—livedo reticularis, renal failure, multiple organ infarction, and death. Cerebral cholesterol embolism was confirmed in three patients with postmortem examination. In another four cases, cholesterol emboli were directly linked to vascular procedures. These patients presented with new neurological symptoms and other systemic manifestations of CES following carotid angioplasty, thoracic aortography, coronary artery bypass grafting, and percutaneous coronary intervention. In one instance cholesterol embolic stroke was confirmed on fundoscopy by visualization of Hollenhorst plaques in the retinal artery [24].

Perhaps one of the most interesting concepts is that of spontaneous cholesterol embolization from aortic or carotid plaques as described in three case studies. These presentations can be insidious as neurological deficits accumulate over time from multiple, discreet showers of emboli. One case describes a patient with a two-year history of behavioral changes and gait deterioration who presented acutely with right arm weakness. She was found to have a dissecting aortic arch aneurysm with a heavy burden of ulcerated cholesterol plaque. Postmortem examination revealed cholesterol emboli in multiple organs including bilateral lacunar infarctions in the brain. This case demonstrates that lacunar infarction may be due to an embolic source in some cases, especially when the patient is normotensive [25].

### 6.4. Treatment

At present, there is no specific therapy for CES. Treatment revolves around supportive care and prevention of further cholesterol embolism. There is some evidence that statin therapy reduces the occurrence of CES [26], although no randomized trials have been done to support this. There is no evidence to support antiplatelet or anticoagulation in the treatment or prevention of CES; however, patients with vascular risk factors should be treated appropriately.

## 7. Intravascular Embolic Debris and Aortic Atherosclerosis

### 7.1. Background and Incidence

Intravascular debris commonly becomes dislodged during invasive vascular procedures, putting the patient at risk of cerebral infarction. Multiple studies have used distal filters to collect this debris and analyze its volume and composition. Embolic debris has been recovered from filters in 58–80% of patients undergoing carotid stenting [22, 27] and 75% of patients undergoing transaortic valve replacement (TAVR) [23], although only around 2% of patients present with clinical symptoms of stroke following these procedures. Material caught in the distal protection filters included thrombus, atherosclerotic plaque, calcified material, cholesterol crystals, and valve leaflets [22, 23, 27] (Figure 1). While the phenomenon of debris embolism is quite familiar there were comparatively few reports (*n* = 12) characterizing nonthrombotic debris actually recovered from the cerebral vessels of subjects suffering neurological symptoms. This may increase in the future with increased availability of endovascular therapy.

Aortic atherosclerotic disease is a known risk factor for stroke. The risk of stroke in one year is 10% in patients with severe aortic plaque (>4 mm thickness) [28]. Plaques consisting of fibrin, calcified material, and lipid laden macrophages can embolize spontaneously or as a result of an intravascular procedure. Aortic atherosclerosis is best evaluated by transesophageal echocardiography. Postmortem studies have confirmed a high incidence of aortic plaque disease in patients diagnosed with cryptogenic stroke [29].

### 7.2. Presentation

Our literature review revealed five case reports of periprocedural stroke secondary to dislodgement of intravascular debris. New neurological symptoms were noticed either during the procedure (TAVI, coronary angiography) or immediately upon reversal of the anesthetic (carotid stenting). In three of these cases, the patient was known to have a high burden of aortic atherosclerosis prior to the procedure. Both thrombolysis and embolectomy have been used to successfully treat stroke following these procedures with favourable clinical outcomes [30].

### 7.3. Treatment

Aortic plaque disease presumably confers an increased stroke risk during intravascular procedures, although there have not been studies to confirm this. However, various other factors have been associated with increased risk including advanced age, complexity of the procedure, and number of passes made with the catheter [31]. Paradoxically, smoking was inversely associated with risk of plaque embolization in one study [22]. Currently, no guidance for standard treatment exists for treatment of stroke following intravascular procedures. The nature of the embolus is variable, and some are more amenable than others to thrombolysis. Furthermore, these patients may have a higher bleeding risk. Few case reports confirm the benefit of thrombolysis in these cases and embolectomy may also play an important role in such cases in the future.

## 8. Other Sources

A single case report was identified of fatal cerebral infarction following embolization of nucleus pulposus from an intravertebral disc to the middle cerebral artery following trauma [32]. This has also been described as a cause of spinal infarction and stroke [33].

## 9. Discussion

Cerebral infarction, while most commonly caused by thromboembolism, can be linked to a large number of nonthrombotic sources. Nonthrombotic embolic stroke is an important diagnosis not to miss in certain clinical settings. Because nonthrombotic cerebral embolism is relatively rare, and the emboli are varied, no uniform approach to management and diagnosis exists. Treatment is largely anecdotal and based on individual case reports or small case series. A stroke register may be useful to gather knowledge regarding these less common causes of stroke and allow us to accumulate data on effective treatments.

Certain causes of nonthrombotic stroke such as amniotic fluid embolism and rare tumour embolism will remain uncommon and confined to isolated case reports. However, with the aging population and the rise of interventional medicine, certain nonthrombotic emboli are bound to become more common. For example, fat and cholesterol embolism are most often iatrogenic. These emboli result from common surgical procedures such as plastic surgery and orthopaedic manipulation. Intravascular procedures provide a particular challenge in this area. Stroke is a well-documented complication of CEA, TAVI, and coronary angiography. A multitude of debris can become dislodged intraoperatively ranging from stable aortic plaque to valve leaflets to plastic catheter pieces.

Patients who suffer from cerebral infarction either intra- or postoperatively represent a group that provide a diagnostic and treatment challenge. Patients are often sedated, so it can be difficult to ascertain the exact time of symptom onset and to decipher whether symptoms are due to postoperative delirium and sedation, or acute intracranial pathology. These patients also have increased bleeding risk if they were to receive thrombolytic therapy. Going forward, it will be important to develop risk stratification strategies particularly for patients undergoing intravascular procedures with known severe aortic plaque. There is a need to reduce unnecessary procedures such as elective cosmetic surgery and take more care around administering central catheters and injections. Embolic deflection devices are currently undergoing clinical trials but have been shown to be safe and effective in reducing new cerebral infarctions in small studies [34, 35].

In appropriately selected cases, embolectomy may prove to be the way forward in treatment of cerebral infarction resulting from nonthrombotic emboli. Embolectomy has been proven to improve clinical outcomes in patients with proximal arterial occlusions for up to six hours after onset of cerebral ischemia. In certain cases this time window can be extended to 12 hours [36]. This longer time window and reduced systemic bleeding risk make embolectomy ideal in cases of periprocedural stroke. Furthermore, while thrombolysis is specific to clots consisting of aggregated platelets and fibrin, mechanical retrieval can be used effectively for a variety of materials. Embolus morphology and characteristics may make certain types of debris more difficult to remove.

This paper has previously been presented at the stroke session of the Irish Gerontological Scoiety Annaual Scientific meeting [37].

## Figures and Tables

**Table 1:** Gurd and Wilson criteria for diagnosis of fat embolism.

Major criteria	Minor criteria
Petechiae in vest distribution	Tachycardia (HR >110 bpm)
Hypoxaemia (PaO2 <60 mmHg)	Pyrexia (>38.5 C)
Central Nervous System Depression	Emboli visible in retinal vessels
Pulmonary Oedema	Fat in urine
	Fat in sputum
	Unexplained drop in hematocrit or platelet count
	Increasing ESR

**Table 2:** Causes of cerebral fat embolism (number of case reports with specific etiology identified).

Traumatic	Nontraumatic
Bone fractures, particularly long bones or pelvis (6)	Pancreatitis (2)
Orthopaedic procedures (12)	Sickle cell haemoglobinopathies (6)
Autologous fat injection (10)	Lipid injection (lymphography) (2)
Cardiac surgery (3)	Muscular dystrophy (5)
Pleural irrigation (1)	Hepatic failure/necrosis (2)

## Supplementary Materials

Supplementary Materials Appendix 1: a bibliography of papers reviewed additional to those in the references in the drafting of this manuscript.
