# Supplementary material for: A Systematic Review of the Causes and Management of Nonthrombotic Embolic Stroke of Tissue Origin
Source: Stroke Res Treat. 2018 Apr 24;2018:8092862. doi: 10.1155/2018/8092862 (PMC5941808; doi:10.1155/2018/8092862)
Supplement: Supplementary Materials — Appendix 1: a bibliography of papers reviewed additional to those in the references in the drafting of this manuscript. [file 8092862.f1.docx]

NTES- articles included in systematic review

1. Chanimov M, Ben-Shlomo I, Chayen B, Gurovich V, Friedland M, Cohen ML, et al. Amniotic fluid embolism: a plea for better brain protection. Isr Med Assoc J IMAJ. 2008 Feb;10(2):154–5.
2. Abenhaim HA, Azoulay L, Kramer MS, Leduc L. Incidence and risk factors of amniotic fluid embolisms: a population-based study on 3 million births in the United States. Am J Obstet Gynecol. 2008 Jul 1;199(1):49.e1-49.e8.
3. Woo Y-S, Hong S-C, Park S-M, Cho K-H. Ischemic stroke related to an amniotic fluid embolism during labor. J Clin Neurosci Off J Neurosurg Soc Australas. 2015 Apr;22(4):767–8.
4. Kumar V, Khatwani M, Aneja S, Kapur KK. Paradoxical amniotic fluid embolism presenting before caesarean section in a woman with an atrial septal defect. Int J Obstet Anesth. 2010 Jan;19(1):94–8.
5. Abe M, Kohama A, Takeda T, Ishikawa A, Yamada Y, Kawase Y, et al. Effective intravenous thrombolytic therapy in a patient with cerebral infarction associated with left atrial myxoma. Internal Medicine. 2011;50(20):2401-5.
6. Anda T, Haraguchi W, Miyazato H, Tanaka S, Ishihara T, Aozasa K, et al. Ruptured distal middle cerebral artery aneurysm filled with tumor cells in a patient with intravascular large B-cell lymphoma: Case report. Journal of Neurosurgery. 2008;109(3):492-6.
7. Baek SH, Park S, Lee NJ, Kang Y, Cho KH. Effective mechanical thrombectomy in a patient with hyperacute ischemic stroke associated with cardiac myxoma. J Stroke Cerebrovasc Dis. 2014;23(9):e417-9.
8. Bhatia S, Ku A, Pu C, Wright DG, Tayal AH. Endovascular mechanical retrieval of a terminal internal carotid artery breast tumor embolus. J Neurosurg. 2010;112(3):572-4.
9. Bittira B, Tsang J, Huynh T, Morin JF, Hüttner I. Primary right atrial synovial sarcoma manifesting as transient ischemic attacks. Annals of Thoracic Surgery. 2000;69(6):1949-51.
10. Boukriche Y, Guiti C, Logeart D, Vissuzaine C, Masson C. Papillary fibroelastoma. A rare but treatable cause of ischemic stroke. Revue de Medecine Interne. 2001;22(8):745-8.
11. Brown DV, Faber LP, Tuman KJ. Perioperative Stroke Caused by Arterial Tumor Embolism. Anesthesia and Analgesia. 2004;98(3):806-9.
12. Browne WT, Wijdicks EFM, Parisi JE, Viggiano RW. Fulminant brain necrosis from atrial myxoma showers. Stroke. 1993;24(7):1090-2.
13. Budzilovich G, Aleksic S, Greco A, Fernandez J, Harris J, Finegold M. Malignant cardiac myxoma with cerebral metastases. Surg Neurol. 1979;11(6):461-9.
14. Byon JH, Kwak HS, Chung GH, Jang KY. Acute stroke from tumor embolus in a patient with cardiac sarcoma: Aspiration thrombectomy with Penumbra catheter. Interventional Neuroradiology. 2016;22(1):88-90.
15. Chalmers N, Campbell IW. Left atrial metastasis presenting as recurrent embolic strokes. Br Heart J. 1987;58(2):170-2.
16. Chan KW, Fryer CJ, Fraser GC, Dimmick JE. Sudden cerebral death in malignant presacral teratoma. Med Pediatr Oncol. 1985;13(6):395-7.
17. Chapman AJ, Chase L. Left atrial myxoma. A case report of unusual complication. American Journal of Forensic Medicine and Pathology. 1984;5(1):69-74.
18. Chong JY, Vraniak P, Etienne M, Sherman D, Elkind MS. Intravenous thrombolytic treatment of acute ischemic stroke associated with left atrial myxoma: a case report. J Stroke Cerebrovasc Dis. 2005;14(1):39-41.
19. Córdoba R, Valldeoriola F, Palombo H, Graus F, Tolosa E. Choriocarcinoma presenting as cerebral infarction due to tumor embolism. Neurología (Barcelona, Spain). 1994;9(2):77-8.
20. Deck JH, Lee MA. Mucin embolism to cerebral arteries: a fatal complication of carcinoma of the breast. Can J Neurol Sci. 1978;5(3):327-30.
21. Dimitrović A, Breitenfeld T, Supanc V, Roje-Bedeković M, Butković Soldo S, Vargek-Solter V. Stroke Caused by Lung Cancer Invading the Left Atrium. Journal of Stroke and Cerebrovascular Diseases. 2016.
22. Gandhi AK, Pearson AC, Orsinelli DA. Tumor invasion of the pulmonary veins: a unique source of systemic embolism detected by transesophageal echocardiography. J Am Soc Echocardiogr. 1995;8(1):97-9.
23. Hauser C, Guelpa G, Lopez JL, Janzer RC, Chaubert P, Baumann RP. Cardiac fibrosarcoma diagnosed as cerebral metastasis. Revue Medicale de la Suisse Romande. 1999;119(10):833-7.
24. Horowitz MB, Carrau R, Crammond D, Kanal E. Risks of tumor embolization in the presence of an unrecognized patent foramen ovale: case report. AJNR Am J Neuroradiol. 2002;23(6):982-4.
25. Horsmann C, Hermanns B, Sellhaus B, Fritz H, Kindler J. Tumor embolism as clinical manifestation of a central bronchial carcinoma. History and admission findings. Tumor Diagnostik und Therapie. 2001;22(2):20-4.
26. Hughes SE, Hunter A, Campbell J, Brady A, Herron B, Smyth G, et al. Extraction of tumour embolus following perioperative stroke. Journal of the Neurological Sciences. 2015;353(1-2):172-4.
27. Imaizumi K, Murate T, Ohno J, Shimokata K. Cerebral infarction due to a spontaneous tumor embolus from lung cancer. Respiration. 1995;62(3):155-6.
28. Januzzi Jr JL, Garasic JM, Neilan TG, Gonzalez RG, Stone JR. Case 28-2007: A 68-year-old man with syncope. New England Journal of Medicine. 2007;357(11):1137-45+074.
29. Jeon U, Cho YS, Kim DH, Park SH, Lee SJ, Shin WY, et al. Probable left atrial myxoma presenting as concurrent cerebral and myocardial infarctions. Korean Circulation Journal. 2008;38(11):622-6.
30. Katz MG, Finkelshtein V, Raichman DB, Dekel H, Lampl Y, Sasson L. Surgical resection of left atrial myxoma presenting with acute multiple hemorrhagic cerebral infarctions: a case report. Heart Surg Forum. 2008;11(3):E169-71.
31. Kim CS, Jung HR, Cho KH, Chang HW, Sohn SI, Choi TH, et al. Forced-suction thrombectomy of an arterial tumor embolism due to metastatic melanoma. Arch Neurol. 2012;69(2):272-3.
32. Lee VH, Connolly HM, Brown RD, Jr. Central nervous system manifestations of cardiac myxoma. Arch Neurol. 2007;64(8):1115-20.
33. Lefkovitz NW, Roessmann U, Kori SH. Major cerebral infarction from tumour embolus. Stroke. 1986;17(3):555-7.
34. Lewis JM. Multiple retinal occlusions from a left atrial myxoma. American Journal of Ophthalmology. 1994;117(5):674-5.
35. Martin-Negrier ML, Belleannée G, Vital C, Orgogozo JM. Primitive malignant fibrous histiocytoma of the neck with carotid occlusion and multiple cerebral ischemic lesions. Stroke. 1996;27(3):536-7.
36. Mastroroberto P. Papilloma of the aortic valve [3]. Texas Heart Institute Journal. 1991;18(4):300-1.
37. Mehta R, Schubbert T, Marshall J, Carpenter P. Rational and successful use of carboplatin and albumin-bound paclitaxel in a patient with recurrent metaplastic carcinoma who presented with multi-organ tumor emboli. Clinical Breast Cancer. 2009;9(1):56-9.
38. Mitomi M, Kimura K, Iguchi Y, Hayashida A, Nishimura H, Irei I, et al. A case of stroke due to tumor emboli associated with metastatic cardiac liposarcoma. Internal Medicine. 2011;50(14):1489-91.

39. Moore L, Byard RW. Fatal paradoxical embolism to the left carotid artery during partial resection of Wilms' tumor. Pediatric Pathology. 1992;12(4):551-6.

40. Navi BB, Kawaguchi K, Hriljac I, Lavi E, DeAngelis LM, Jamieson DG. Multifocal stroke from tumor emboli. Archives of Neurology. 2009;66(9):1174-5.

41. O'Neill BP, Dinapoli RP, Okazaki H. Cerebral infarction as a result of tumor emboli. Cancer. 1987;60(1):90-5.

42. Park JH, Seo HS, Park SK, Suh J, Kim DH, Cho YH, et al. Spontaneous systemic tumor embolism caused by tumor invasion of pulmonary vein in a patient with advanced lung cancer. J Cardiovasc Ultrasound. 2010;18(4):148-50.

43. Rachinger J, Trenkler J. Cerebral oncotic aneurysms in embolic cardiac myxoma with delayed neurologic complications. Case report. Klinische Neuroradiologie. 1999;9(4):265-9.

44. Ramachandren TK, Venkataraman K, Hussey K, Ferguson L. Metastatic Angiosarcoma Presenting as Ischemic Anterior Circulation Stroke. Ann Vasc Surg. 2016;31:209.e7-9.

45. Robbin NA, Landless P, Cooper K, Fritz VU. Myxoma in the carotid artery: Myxomatous occlusion of internal carotid artery. Stroke. 1997;28(2):456-8.

46. Saber S, Liewelyn M, Zachariah G, Umachandran V. The complex relationship between cancer and cerebral vascular accidents. International Journal of Stroke. 2015;10:254.

47. Schreffler SM, Paolo WF, Kloss BT. Spontaneous showering of tumor emboli in a patient with advanced primary lung cancer: A case report. International Journal of Emergency Medicine. 2012;5(1).

48. Spaulding Rt, Koumoundouros T, Parker JC, Jr. Metastatic undifferentiated pleomorphic sarcoma causing intraoperative stroke. Ann Clin Lab Sci. 2013;43(2):172-5.

49. Sun MC, Tai HC, Lee CH. Intravenous Thrombolysis for Embolic Stroke due to Cardiac Myxoma. Case Rep Neurol. 2011;3:21-6.

50. Tasi SH, Juan CJ, Dai MS, Kao WY. Trousseau's syndrome related to adenocarcinoma of the colon and cholangiocarcinoma. Eur J Neurol. 2004;11(7):493-6.

51. Towfighi J, Simmonds MA, Davidson EA. Mucin and fat emboli in mucinous carcinomas. Cause of hemorrhagic cerebral infarcts. Archives of Pathology and Laboratory Medicine. 1983;107(12):646-9.

52. Umemura S, Kishino D, Tabata M, Kiura K, Hotta K, Nishii K, et al. Systemic tumor embolism mimicking gefitinib ('IRESSA')-induced interstitial lung disease in a patient with lung cancer. Internal Medicine. 2005;44(9):979-82.

53. Vega RA, Chan JL, Anene-Maidoh TI, Grimes MM, Reavey-Cantwell JF. Mechanical thrombectomy for pediatric stroke arising from an atrial myxoma: case report. Journal of neurosurgery Pediatrics. 2015;15(3):301-5.

54. Veinot JP, Katz AS, Conte FJ. Fibroelastoma and embolic stroke [1] (multiple letters). Circulation. 1999;99(20):2709.

55. Abend NS, Levine JM. Hypodense middle cerebral artery with fat embolus. Neurocrit Care. 2007;6(2):147-8.

56. Adams JH, Graham DI, Mills E, Sprunt TG. Fat embolism and cerebral infarction after use of methylmethacrylic cement. Br Med J. 1972;3(5829):740-1.

57. Alfonso DT, Toussaint RJ, Alfonso BD, Strauss EJ, Steiger DT, Di Cesare PE. Nonsurgical complications after total hip and knee arthroplasty. Am J Orthop (Belle Mead NJ). 2006;35(11):503-10.

58. Alobeidi F, Inusa BPD, Singh RR, U-King-Im JM. Cerebral microhaemorrhages secondary to fat embolus syndrome in sickle cell disease. Postgraduate Medical Journal. 2015;91(1071):55-6.

59. Barak M, Kabha M, Norman D, Soudry M, Kats Y, Milo S. Cerebral microemboli during hip fracture fixation: A prospective study. Anesthesia and Analgesia. 2008;107(1):221-5.

60. Battle C, Dow A, Timperely J. Fatal systemic embolism following revision of hip arthroplasty: A complication of pulmonary arteriovenous malformation. Journal of the Intensive Care Society. 2013;14(2):165-8.

61. Brown WR, Moody DM, Challa VR. Cerebral fat embolism from cardiopulmonary bypass. Journal of Neuropathology and Experimental Neurology. 1999;58(2):109-19.

62. Byrick RJ. Causes of brain injury during orthopedic surgery. Canadian Journal of Anesthesia. 2004;51(9):867-70.

63. Carmona R, Tuan A, Hughes TH, Dorros SM. Cerebral fat embolism syndrome following revision of right total hip arthroplasty. BMJ Case Reports. 2014.

64. Chiba M, Imaizumi T, Honma T, Niwa J, Makabe T, Moriyama R, et al. Dynamic changes in magnetic resonance diffusion-weighted and fluid-attenuated images of cerebral fat embolism: A case report. Brain and Nerve. 2002;54(8):693-6.

65. Cox G, Tzioupis C, Calori GM, Green J, Seligson D, Giannoudis PV. Cerebral fat emboli: a trigger of post-operative delirium. Injury. 2011;42 Suppl 4:S6-s10.

66. Danesh-Meyer HV, Savino PJ, Sergott RC. Case reports and small case series: ocular and cerebral ischemia following facial injection of autologous fat. Arch Ophthalmol. 2001;119(5):777-8.

67. Feinendegen DL, Baumgartner RW, Vuadens P, Schroth G, Mattle HP, Regli F, et al. Autologous fat injection for soft tissue augmentation in the face: A safe procedure? Aesthetic Plastic Surgery. 1998;22(3):163-7.

68. Goenka N, Ropper AH. Cerebral fat embolism. New England Journal of Medicine. 2012;367(11):1045.

69. Grunwald IQ, Bose A, Struffert T, Romeike BF, Politi M, Reith W, et al. Liposuction in mind. Archives of Neurology. 2009;66(6):800-1.

70. Hong DK, Seo YJ, Lee JH, Im M. Sudden visual loss and multiple cerebral infarction after autologous fat injection into the glabella. Dermatologic Surgery. 2014;40(4):485-7.

71. Hu J, Chen W, Wu Y, Chen K, Luo C, Liang Y, et al. Middle cerebral artery occlusion following autologous bitemporal fat injection. Neurology India. 2011;59(3):474-5.

72. Kallina IC, Probe R. Paradoxical fat embolism after intramedullary rodding: a case report. J Orthop Trauma. 2001;15(6):442-5.

73. Kuo KH, Pan YJ, Lai YJ, Cheung WK, Chang FC, Jarosz J. Dynamic MR imaging patterns of cerebral fat embolism: A systematic review with illustrative cases. American Journal of Neuroradiology. 2014;35(6):1052-7.

74. Kusumoto S, Imamura A, Watanabe K. Case report: the incidental lipid embolization to the brain and kidney after lymphography in a patient with malignant lymphoma: CT findings. Clin Radiol. 1991;44(4):279-80.

75. Lee CM, Hong IH, Park SP. Ophthalmic artery obstruction and cerebral infarction following periocular injection of autologous fat. Korean J Ophthalmol. 2011;25(5):358-61.

76. Lee KM, Kim EJ, Jahng GH, Chang DI. Magnetic resonance findings in two episodes of repeated cerebral fat embolisms in a patient with autologous fat injection into the face. Journal of Korean Neurosurgical Society. 2012;51(5):312-5.

77. Liu C, Yadava R, Esakji M, Al-Saadi S, Thakkar C, Syed H. Bihemispheric cortical fat emboli following revision surgery of previous total hip replacement. International Journal of Stroke. 2014;9:56.

78. Malik AM. Portable head computed tomography in the diagnosis of cerebral fat embolism secondary to cardiac surgery. Neurohospitalist. 2012;2(4):154-5.

79. McAdam LC, Rastogi A, MacLeod K, Douglas Biggar W. Fat Embolism Syndrome following minor trauma in Duchenne muscular dystrophy. Neuromuscular Disorders. 2012;22(12):1035-9.

80. Mijalski C, Lovett A, Mahajan R, Sundararajan S, Silverman S, Feske S. Cerebral Fat Embolism: A Case of Rapid-Onset Coma. Stroke. 2015;46(12):e251-3.

81. Moreno Garcia MP, Gil Alzueta MC, Herrera Isasi MC, Zandio Amorena B, Erro Aguirre ME. Acute encephalopaty secondary to fat embolism. Cerebrovascular Diseases. 2010;29:93.

82. Price MD, Kanake P, Talmor D. Paradoxical Embolus after Multiple Trauma Resulting in a Cerebrovascular Accident. Anesthesia and Analgesia. 2004;98(4):1121-3.

83. Rafik R, Hachimi MA, Ouarssani A, Atoini F, Rouimi A. A rare cause of cerebral ischemic stroke: Cerebral fat embolism. Revue Neurologique. 2012;168(3):298-9.

84. Ramachandiran N, Raniga S, Al Kindi S, Dennison J, Al Farsi K, Al Busaidi M, et al. Non-traumatic cerebral fat embolism in sickle cell disease. Neurology. 2016;86(16).

85. Rathmalgoda CM, Aravind A, Aweid B, Kar A. Massive fat embolism causing malignant MCA Syndrome. Cerebrovascular Diseases. 2015;39:330.

86. Rudrappa M, Khasawneh K, Kokatnur L, Colaco B, Colaco C, Mittadodla P. Rare care of coma: Cerebral fat embolism following arthroplasty. Critical Care Medicine. 2014;42(12):A1648-A9.

87. Takinami Y. Two cases of cerebral infarction caused by fat embolism during orthopedic bone surgeries. Japanese Journal of Anesthesiology. 2009;58(8):993-6.

88. Thaunat O, Thaler F, Loirat P, Decroix JP, Boulin A. Cerebral fat embolism induced by facial fat injection [21]. Plastic and Reconstructive Surgery. 2004;113(7):2235-6.

89. Toledano S, Zyss J, Gerber S, Rodallec M, Zuber M. Fat emboli responsible for ischemic stroke in reconstructive eye surgery. Journal of Neurology. 2010;257(11):1927-8.

90. Van Oostenbrugge RJ, Freling G, Lodder J, Lalisang R, Twijnstra A. Fatal stroke due to paradoxical fat embolism [1]. Cerebrovascular Diseases. 1996;6(5):313-4.

91. Wang DW, Yin YM, Yao YM. Internal and external carotid artery embolism following facial injection of autologous fat. Aesthet Surg J. 2014;34(8):Np83-7.

92. Wang YC, Fu JH, Lai PH. Teaching NeuroImages: Hypodense artery sign in acute cerebral infarction by contrast-enhanced CT. Neurology. 2009;73(4):e16.

93. Wegener K, Bolgert F, Pierrot-Deseilligny C. A case of cerebral fat embolism demonstrating no pathophysiological involvement of lung dysfunction. Eur Neurol. 1999;42(1):65-6.

94. Whalen LD, Khot SP, Standage SW. High-dose rosuvastatin treatment for multifocal stroke in trauma-induced cerebral fat embolism syndrome: A case report. Pediatric Neurology. 2014;51(3):410-3.

95. Yeon HB, Ramappa A, Landzberg MJ, Thornhill TS. Paradoxic cerebral embolism after cemented knee arthroplasty: a report of 2 cases and prophylactic option for subsequent arthroplasty. J Arthroplasty. 2003;18(1):113-20.

96. Yilmaz A, Kurt S, Sarikaya B, Firat MM, Ocal S, Yeginsu A. Recurrent cerebral fat embolism due to pleural irrigation: Fat density lesions on CT. A case report. Neuroradiology Journal. 2007;20(3):287-90.

97. Yoon SS, Chang DI, Chung KC. Acute fatal stroke immediately following autologous fat injection into the face. Neurology. 2003;61(8):1151-2.

98. Cholesterol emboli. Consultant. 2004;44(4):583.

99. Balla JI, Howat JM, Walton JN. Cholesterol emboli in retinal arteries. Journal of neurology, neurosurgery, and psychiatry. 1964;27:144-8.

100. Beal MF, Williams RS, Richardson Jr EP, Fisher CM. Cholesterol embolism as a cause of transient ischemic attacks and cerebral infarction. Neurology. 1981;31(7):860-5.

101. Chebut O, Medeiros De Bustos E, Moulin T. The story of a sleeping monster - A case report; one of the new treatments that changed the face of modern neurology can be put in danger. European Journal of Neurology. 2011;18:112.

102. Gordhan A, Ray W. Retinal cholesterol embolism and visual loss post carotid angioplasty and stenting. American Journal of Case Reports. 2008;9:159-62.

103. Heinzlef O. Neurological manifestations of cholesterol embolization. Sang Thrombose Vaisseaux. 1998;10(3):144-50.

104. Ichinoe M, Mikami T, Ujiie S, Suzuki K, Okayasu I. Heparin-induced thrombocytopenia with multiple organized thrombi accompanied by unusual cholesterin deposition: autopsy case after long-term follow up. Pathol Int. 2009;59(10):757-61.

105. Laloux P, Brucher JM. Lacunar infarctions due to cholesterol emboli. Stroke. 1991;22(11):1440-4.

106. Müller I, Bockholt A, Ferbert A. Multiple cerebral infarction from spontaneous synchronous cholesterol emboli. Nervenarzt. 2002;73(4):371-5.

107. Nakamoto S, Kaneda T, Inoue T, Matumoto T, Onoe M, Kitayama H, et al. Disseminated cholesterol embolism after coronary artery bypass grafting. J Card Surg. 2001;16(5):410-3.

108. Nakamura M, Tokura Y. Systemic cholesterol embolization syndrome in a patient positive for anti-cardiolipin antibody. Dermatoendocrinol. 2010;2(2):58-9.

109. Pascual M, Baumgartner JM, Bounameaux H. Stroke secondary to multiple spontaneous cholesterol emboli. Vasa. 1991;20(1):74-7.

110. Rapp JH, Pan XM, Neumann M, Hong M, Hollenbeck K, Liu J. Microemboli composed of cholesterol crystals disrupt the blood-brain barrier and reduce cognition. Stroke. 2008;39(8):2354-61.

111.Tekin B, Sariçam MH, Özgen Z, Demirkesen C, Komesli Z, Arikan IH. A 60-year-old man with bilateral painful reticular patches of violaceous colour on the soles. Marmara Medical Journal. 2013;26(3):178-80.

112. Wolf I, Mouallem M. Multiple organ damage due to cholesterol embolization. Harefuah. 1999;137(9):375-8, 431.

113. Yadav P, Hyde E, Thimmapuram J, McKeague M. Rare case of isolated partial occulomotor nerve palsy post cardiac catheterization. Journal of General Internal Medicine. 2010;25:S543.

114. Yang S, Dong H, Yao L, Gu X, Shi R, Yao K, et al. More severe brain damage found in cholesterol-embolic stroke compared with clot-embolic stroke. Arteriosclerosis, Thrombosis, and Vascular Biology. 2015;35.

115. Yang S, Dong H, Yao L, Gu X, Zhao C, Zhang W. Pathological differences of ischemic stroke caused by cholesterol versus clot embolization. Stroke. 2015;46.

116. Young BWM, Gunathilagan G, Thomas G. Cholesterol emboli syndrome (CES) following stroke thrombolysis and anticoagulation with warfarin. Cerebrovascular Diseases. 2012;33:169.
117. Divani AA, Berezina TL, Zhou J, Pakdaman R, Suri MF, Qureshi AI. Microscopic and macroscopic evaluation of emboli captured during angioplasty and stent procedures in extracranial vertebral and internal carotid arteries. J Endovasc Ther. 2008;15(3):263-9.

118. Khatri P, Kasner SE. Ischemic strokes after cardiac catheterization: opportune thrombolysis candidates? Arch Neurol. 2006;63(6):817-21.

119. Koike R, Oku T, Satoh H, Fukuda S, Minohara S, Sawada Y, et al. Multiple cerebral infarction following coronary artery bypass grafting for the patient with calcified aorta--a case report. Rinshō kyōbu geka = Japanese annals of thoracic surgery. 1989;9(4):396-9.

120. Matsukawa H, Fujii M, Uemura A, Suzuki K, Yamamoto D, Takahashi O, et al. Pathology of embolic debris in carotid artery stenting. Acta Neurologica Scandinavica. 2015;131(4):197-202.

121. Matsuyama TA, Ishibashi-Ueda H, Ikeda Y, Nagatsuka K, Miyashita K, Amaki M, et al. Critical multi-organ emboli originating from collapsed, vulnerable caseous mitral annular calcification. Pathol Int. 2012;62(7):496-9.

122. McKibbin DW, Gott VL, Hutchins GM. Fatal cerebral atheromatous embolization after cardiopulmonary bypass. J Thorac Cardiovasc Surg. 1976;71(5):741-5.

123. Pinero P, Gonzalez A, Martinez E, Mayol A, Rafel E, Gonzalez-Marcos JR, et al. Volume and composition of emboli in neuroprotected stenting of the carotid artery. AJNR Am J Neuroradiol. 2009;30(3):473-8.

124. Saric M, Kronzon I. Aortic atherosclerosis and embolic events. Current Cardiology Reports. 2012;14(3):342-9.

125. Van Laanen JHH, Hendriks JM, Verhagen HJM, Van Beusekom HMM. Quantity, particle size, and histologic composition of embolic debris collected in a distal protection filter after carotid angioplasty and stenting: Correlation with patient characteristics, timing of carotid artery stenting, and procedural details. Journal of Thoracic and Cardiovascular Surgery. 2013;146(2):492-5.

126. Van Mieghem NM, Schipper MEI, Ladich E, Faqiri E, Van Der Boon R, Randjgari A, et al. Histopathology of embolic debris captured during transcatheter aortic valve replacement. Circulation. 2013;127(22):2194-201.

127. Velasco A, Mosimann PJ. Distal cerebral protection device filled with calcified plaque debris after carotid stenting. JACC Cardiovasc Interv. 2013;6(4):e22-3.

128. Zakhari N, Castillo M, Torres C. Unusual Cerebral Emboli. Neuroimaging Clinics of North America. 2016;26(1):147-63.

129. Geng J, Tian HY, Zhang YM, He S, Ma Q, Zhang JB, Liu Y, Tian H, Zhang D, Meng Y. Paradoxical embolism: A report of 2 cases. Medicine (Baltimore). 2017; 96: e7332.

130. Ohshima K, Tsujii Y, Sakai K, Oku H, Morii E. Massive tumor embolism in the abdominal aorta from pulmonary squamous cell carcinoma: Case report and review of the literature. Pathol Int. 2017; 67: 467-471.

131. Saito Y, Aizawa Y, Monno K, Nagashima K, Kurokawa S, Osaka S, Akimoto T, Kamei S, Tanaka M, Hirayama A. Small, smooth, nonmobile cardiac myxoma detected by transesophageal echocardiography following recurrent cerebral infarction: a case report. J Med Case Rep. 2017; 11: 131.

132. Singu T, Inatomi Y, Yonehara T, Ando Y. Calcified Amorphous Tumor Causing Shower Embolism to the Brain: A Case Report with Serial Echocardiographic and Neuroradiologic Images and a Review of the Literature. J Stroke Cerebrovasc Dis. 2017 May;26(5):e85-e89.

133. Dimitrović A, Breitenfeld T, Supanc V, Roje-Bedeković M, Butković Soldo S,Vargek-Solter V. Stroke Caused by Lung Cancer Invading the Left Atrium. J Stroke Cerebrovasc Dis. 2016; 25:e66-e68.

134. Biraschi F, Diana F, Alesini F, Guidetti G, Peschillo S. Effective ADAPT Thrombectomy in a Patient with Acute Stroke due to Cardiac Papillary Elastofibroma: Histological Thrombus Confirmation. J Stroke Cerebrovasc Dis. 2016; 25:e185-7.

135. Zander T, Maynar J, López-Zárraga F, Herrera R, Timiraos-Fernández JJ, Saraceni A, Maynar M. Mechanical thrombectomy in patients with tumour-related ischaemic stroke. Interv Neuroradiol. 2016 Dec;22(6):705-708.

136. Coughlan JJ, Fleck R, O'Connor C, Crean P. Mechanical thrombectomy of embolised native aortic valve post-TAVI. BMJ Case Rep. 2017 Feb 9;2017.


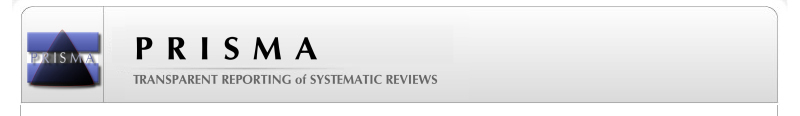
**PRISMA 2009 Flow Diagram**

Studies included in qualitative synthesis (case reports and series)
Tumour (n = 60)

Fat (n = 43)

Cholesterol (n = 19)

Endovascular debris

(n = 12)

Amniotic fluid (n = 4)

Full-text articles excluded, with reasons
Not in English (n = 10 )

Not relevant (n = 12 )

Full-text articles assessed for eligibility
(n = 158 )

Records excluded
(n = 639 )

Records screened
(n = 799 )

Records after duplicates removed
(n = 799 )

Additional records identified through other sources
(n = 0)

## Identification

## Eligibility

## Included

## Screening

Records identified through database searching
(n = 1020 )
